# Shift work schedules alter immune cell regulation and accelerate cognitive impairment during aging

**DOI:** 10.1186/s12974-024-03324-z

**Published:** 2025-01-08

**Authors:** Karienn A. de Souza, Morgan Jackson, Justin Chen, Jocelin Reyes, Judy Muayad, Emma Tran, William Jackson, M. Karen Newell-Rogers, David J. Earnest

**Affiliations:** 1 Department of Neuroscience and Experimental Therapeutics, School of Medicine, Texas A&M Health Science Center, Bryan, TX 77807-3260 USA; 2 Department of Medical Physiology, College of Medicine, Texas A&M Health Science Center, Bryan, TX 77807-3260 USA; 3 Department of NExT, Texas A&M Health Science Center, 8447 State Highway 47, 2004 MREB, Bryan, TX 77807-3260 USA

**Keywords:** Circadian rhythm dysregulation, Activity rhythm, Barnes maze, Cognition, Adaptive immune cell, B cells, T cells, Microglia, Aging, Mice

## Abstract

**Background:**

Disturbances of the sleep-wake cycle and other circadian rhythms typically precede the age-related deficits in learning and memory, suggesting that these alterations in circadian timekeeping may contribute to the progressive cognitive decline during aging. The present study examined the role of immune cell activation and inflammation in the link between circadian rhythm dysregulation and cognitive impairment in aging.

**Methods:**

C57Bl/6J mice were exposed to shifted light-dark (LD) cycles (12 h advance/5d) during early adulthood (from ≈ 4-6mo) or continuously to a “fixed” LD12:12 schedule. At middle age (13-14mo), the long-term effects of circadian rhythm dysregulation on cognitive performance, immune cell regulation and hippocampal microglia were analyzed using behavioral, flow cytometry and immunohistochemical assays.

**Results:**

Entrainment of the activity rhythm was stable in all mice on a fixed LD 12:12 cycle but was fully compromised during exposure to shifted LD cycles. Even during “post-treatment” exposure to standard LD 12:12 conditions, re-entrainment in shifted LD mice was marked by altered patterns of entrainment and increased day-to-day variability in activity onset times that persisted into middle-age. These alterations in light-dark entrainment were closely associated with dramatic impairment in the Barnes maze test for the entire group of shifted LD mice at middle age, well before cognitive decline was first observed in aged (18-22mo) animals maintained on fixed LD cycles. In conjunction with the effects of circadian dysregulation on cognition, shifted LD mice at middle age were distinguished by significant expansion of splenic B cells and B cell subtypes expressing the activation marker CD69 or inflammatory marker MHC Class II Invariant peptide (CLIP), differential increases in CLIP+, 41BB-Ligand+, and CD74 + B cells in the meningeal lymphatics, alterations in splenic T cell subtypes, and increased number and altered functional state of microglia in the dentate gyrus. In shifted LD mice, the expansion in splenic B cells was negatively correlated with cognitive performance; when B cell numbers were higher, performance was worse in the Barnes maze. These results indicate that disordered circadian timekeeping associated with early exposure to shift work-like schedules alone accelerates cognitive decline during aging in conjunction with altered regulation of immune cells and microglia in the brain.

## Introduction

Aging is the primary risk factor for the progressive cognitive decline in the elderly and in Alzheimer’s disease-related dementias (ADRDs). The hippocampus and basal forebrain (BF) are responsible for explicit and spatial memory [1] and pathophysiological changes, such as neurodegeneration and activation of microglia and other immune cells, in these brain regions have been linked to age-related cognitive deficits [2–5]. Cognitive tests that probe the integrity of these brain regions have historically provided the diagnostic focus for dementia during aging [6–8]. However, there is increasing evidence that atypical behavioral symptoms often occur early, prior to the development and progression of the cognitive decline and as such, may provide early biomarkers of dementia or may even contribute to age-related cognitive deficits.

Among the other neurobehavioral changes linked to progressive memory loss during aging and in ADRDs, pronounced alterations in the sleep-wake cycle and circadian rhythms typically accompany, or even precede, the hallmark cognitive decline [9, 10]. While circadian and sleep-wake disturbances are observed even during normal aging in healthy individuals, elderly patients with mild to severe dementia are commonly distinguished by delayed sleep-wake patterns in which both bedtime and wake times occur later in the day and by irregular sleep-wake rhythm disorder with high variability in the timing of sleep onset and wakefulness [10–13]. In this regard, we have used the C57Bl/6J mouse model to identify specific changes in the circadian rhythm of activity that are age-dependent and that occur early in the development and progression of age-related cognitive impairment. Our published data indicates that aged C57Bl/6J mice show irregular sleep-wake patterns similar to those reported for elderly populations, but these changes in circadian rhythmicity are first observed at middle age (13-14mo), well before the learning and memory deficits found in aged animals [14]. Specifically, the activity rhythms of middle-aged and aged mice during light-dark (LD) entrainment showed daily onsets of activity that were significantly delayed and highly variable in their timing between successive days relative to the young cohort. Consistent with human studies on the implications of circadian dysregulation in age-related dementia and ADRDs, the variability in the daily onsets of activity was inversely correlated with the cognitive index across all ages of learning-impaired mice, such that when onset variability was higher, cognitive performance in the Barnes maze was lower.

The implications of these unstable patterns of circadian entrainment in cognitive decline during aging are unclear, but it is noteworthy that circadian rhythm disturbances are thought to be contributing factors in other neurodegenerative diseases such as Parkinson’s disease [15, 16]. Because alterations in circadian entrainment of the activity rhythm effectively differentiated impaired from unimpaired learners across all age groups of mice [14], it is possible that this circadian dysregulation may shift the progression and directly “drive” further age-related deficits in cognition. Thus, the primary objective of this study was to determine the extent to which circadian dysregulation, independent of aging and other risk factors for dementia, contributes to the progression of cognitive impairment. Experiments utilized a unique model, combining an established light-dark (LD) cycle shifting paradigm with our novel analysis of cognitive aging in C57Bl/6J mice, to examine the long-term effects of circadian rhythm dysregulation alone on cognitive function during aging. Because circadian rhythm dysregulation promotes pro-inflammatory responses of the immune system [17, 18] and immune/inflammatory processes have been implicated in the pathophysiology of dementia [19–22], we also determined whether the impact of these shift work-like schedules on cognitive function is coupled with fundamental alterations in the activation of microglia and other immune cells that can modulate inflammatory responses. Our results indicate that exposure to shifted LD cycles during early adulthood induced long-term alterations in the functional state of hippocampal microglia together with an expansion of B cells and a reduction in regulatory T cells (Treg), all of which have been implicated in chronic inflammatory syndromes, autoimmunity, cognitive decline during aging and ADRDs [21, 22].

## Materials and methods

## Animals

Adult male and female C57Bl/6 mice were purchased from the Jackson Laboratory (JAX stock #0664) and maintained in the AAALAC-accredited vivarium at the Texas A&M University Health Science Center. All animals were maintained in vivarium rooms under controlled temperature (22–25 °C) and lighting (LD 12:12) conditions with food (standard mouse chow) and water available *ad libitum*. All animal experiments were performed in accordance with the National Institutes of Health Guide for the Care and Use of Laboratory Animals. Animal procedures used in this study were conducted in compliance with Animal Use Protocol 2022 − 0211 as reviewed and approved by the Institutional Animal Care and Use Committee at Texas A&M University.

**Fig. 1:**
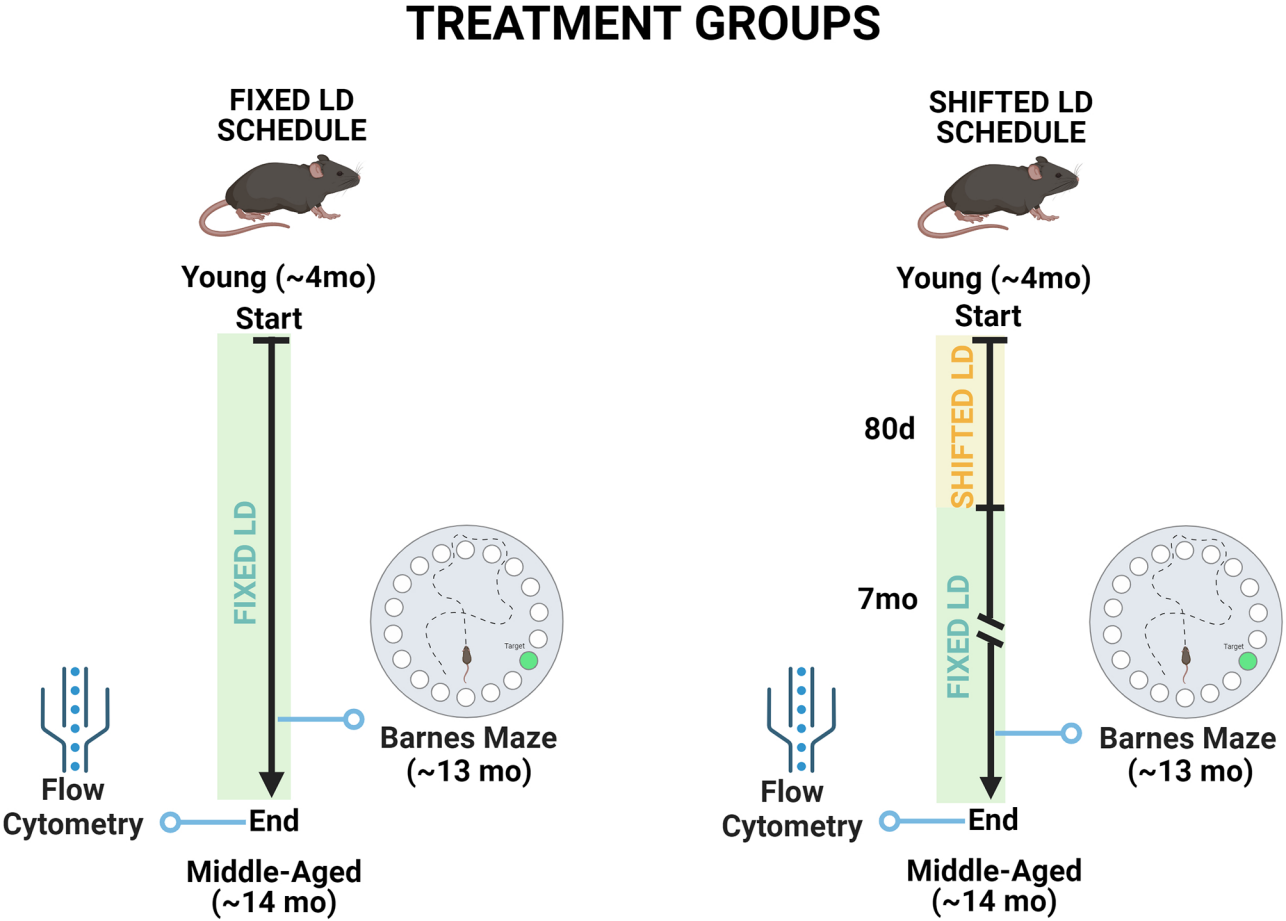
LD treatment groups and experimental design. Mice were separated into two cohorts: (1) control group (left) was maintained throughout on a fixed LD 12:12 schedule and (2) experimental treatment group (right) was exposed to shifted LD cycles (12 h advance/5d) for 80 days and then was placed back on the same standard LD 12:12 cycle. At middle age (13mo), both groups were assessed in the Barnes maze and euthanized at 14 months for flow cytometry analysis. (Created with BioRender.com)

To analyze the effects of circadian dysregulation, experiments used a chronic light-dark (LD) cycle shift paradigm that has been shown to be effective in desynchronizing circadian rhythms and in inducing pro-inflammatory responses of immune cells, leading to a persistent inflammatory condition [17, 18, 23]. After baseline acclimation under standard LD 12:12 conditions (lights-on at 0800 h; light intensity = 110–170 lx at 500–580 nm) for about 2 weeks, young C57Bl/6 mice (≈ 3mo) were randomly divided into 2 groups and exposed for 80 days to either the same “fixed” LD 12:12 cycle (*n* = 20) or to a “shifted” LD 12:12 cycle (*n* = 16). During exposure to the shifted LD paradigm (Fig. 1A), lights-on was advanced by 12 h (at 2000 h) every 5 days and these shifts in the LD cycle were repeated for 8 full cycles. At the conclusion of experimental LD cycle manipulations (“treatment period”), animals (~ 7mo) in both groups were exposed to the same standard LD 12:12 schedule (lights-on at 0800 h) for ~ 7 additional months (“post-treatment period”). Then following completion of behavioral assays, all animals were anesthetized early in the 12-hour photoperiod (0900–1200 h) with isoflurane and tissues were collected for flow cytometry and immunohistochemistry analyses.

### Analysis of wheel-running activity

To confirm the long-term effects of circadian dysregulation on the rhythm of wheel-running behavior, a separate cohort of mice were housed individually in cages equipped with running wheels and divided at ~ 3mo of age into two treatment groups exposed to fixed (*n* = 7) or shifted (*n* = 7) LD cycles as described previously. Wheel-running activity was continuously recorded, stored in 10-minute bins, graphically depicted in actograms, and analyzed using ClockLab data collection and analysis software (ActiMetrics, Evanston, IL). Entrainment and qualitative parameters of the activity rhythm in mice exposed to fixed or shifted LD cycles were measured at middle age over the same interval (40 days) following exposure to experimental LD cycles when both groups were exposed to the same standard LD 12:12 cycle. During LD entrainment, the onset of activity for a given cycle was identified as the first bin during which an animal attained 10% of peak running-wheel revolutions (i.e., intensity). To measure phase angle of entrainment (Ψ), least squares analyses was used to establish a regression line through the daily onsets of activity during the period of entrainment (40 days), and then the number of minutes before (positive) or after (negative) the time of lights-off in the LD cycle (2000 h) was determined for each animal. Total daily activity was calculated by averaging the number of wheel revolutions per 24 h over the interval of analysis. Group differences were established by separately analyzing these entrainment and qualitative parameters of the activity rhythm during the LD “treatment” (fixed, shifted) phase, and the subsequent “post-treatment” phase of the study when both groups were exposed to the same fixed LD cycle.

### Behavioral assays

#### Barnes maze

To assess the effects of environment-induced circadian dysregulation on cognition in relation to normal aging, Barnes maze data from middle-aged (13mo) fixed (*n* = 20, 9 males and 11 females) and shifted (*n* = 16, 6 males and 10 females) LD mice in this study was compared with our published observations from aged (18-22mo; *n* = 29, 14 males and 15 females) C57Bl/6 mice that were constantly maintained on a standard LD 12:12 cycle (lights-on at 0800 h) [14]. The Barnes circular platform maze is a 91.44 cm diameter circular platform on a 1.4 m stand with 20 evenly spaced 5.08 cm diameter holes around the circumference, where a black box (escape tunnel) was placed underneath one of the holes (San Diego Instruments, CA). Four bright lights were positioned above the maze as an aversive stimulus to cause the mice to seek out the escape box using spatial cues. Between each trial, the table and escape box were cleaned with 70% ethanol, and video was acquired using a Color GigE camera (model: acA1300-30gc). Data were quantified using Ethovision XT 16 video tracking software (Noldus, Leesburg, VA).

Barnes maze testing was performed during the early portion of the 12-hour photoperiod (0900–1300 h) as described previously [14]. Our protocol consists of habituation, acquisition training (learning) and probe testing (memory).

*Habituation.* During habituation, mouse was placed on the table for 5 min and allowed to explore the maze without an escape box, under dim lights. Next, mice were placed in a 2 L transparent glass beaker, under aversive lighting. After 1 min, the mouse was gently guided to the escape box and lights are turned off.

*Learning training trials.* On all subsequent trials, the animals were placed into the center of the table under a dark container for 30 s before the container was lifted and the mouse was allowed to navigate the maze using spatial cues under aversive lighting. Each mouse was allowed 180 s to locate and enter the escape box per trial. If the mouse was unable to locate the escape hole after 180 s, it was gently guided to the correct hole location and allowed to enter the escape box. Once the mouse entered the escape box (either guided or on their own), it remained in the box for one minute before returning to its home cage. Each day, the animal was subjected to 4 trials spaced 15 min apart for a total of sixteen learning training trials, for 4 days.

*Probe Trial.* Seventy-two hours after learning trials, the mouse is given a single probe trial, in which escape box is not available. The animal’s search behavior is analyzed for 180 s, after which the mouse is removed and placed back into its holding cage.

*Search Strategies and Cognitive Index.* Detailed description of search strategies is provided in Souza et al. [14]. In brief, the hippocampal-dependent strategies are: direct (no error; score = 1), corrected (searched + or – 1 immediate hole, score = 0.75), focused (searched + or – 3 immediate holes, score = 0.5) and long correction (mouse searches across the target and immediately corrects toward correct hole, score = 0.5). Non-hippocampal strategies include the serial search (animal methodically searches holes one by one, score = 0.25), random (search without a clear strategy and target hole identified by chance, score = 0), and failure (animal searches but does not find the target, score = 0). Scores were summed to produce the Cognitive Index.

### Flow cytometry and immune profiling

To compare the effects of circadian dysregulation and normal aging on immune cell activation, immune profiling using flow cytometry was performed on single cell suspensions [24] of spleen-derived lymphocytes from shifted LD mice at middle age (~ 14mo; *n* = 14, 6 males and 8 females) following exposure to a standard LD 12:12 cycle, and from groups of middle-aged (~ 14mo; *n* = 12, 7 males and 5 females) and aged (18-22mo; *n* = 13, 8 males and 5 females) mice that were continuously maintained on fixed LD cycles. Parallel analyses were performed on lymphocytes extracted from the cranial meninges of fixed (*n* = 12, 7 males and 5 females) and shifted (*n* = 16, 8 males and 8 females) LD mice at middle age (~ 14mo). Isolated lymphocytes from the spleen and cranial meninges were treated with Ammonium-Chloride-Potassium (ACK) Lysis Buffer to lyse red blood cells, resuspended in phosphate-buffered saline (PBS) with 3% heat-inactivated Fetal Bovine Serum (FBS), and then incubated with FC block to prevent non-specific cell staining. Using our previously established procedures for flow cytometry [25], cells were stained with the following fluorochrome-conjugated antibodies: Ghost Dye Red 780 Viability stain, FITC CLIP (15G4; Santa Cruz Biotechnology, Inc., Dallas, TX), BV421 CD19, BV510 CD90.2, Alexa Fluor 700 CD4, BV 785 CD69, PE 41BBL, PerCP MHCII(IA-IE), Alexa Fluor 647 CD74, PE CD44, BV 421 CD62L, APC CD25 and PE FoxP3 (Biolegend, San Diego, CA). Staining for subsets of T cells, B cells, MHCII + or CLIP + lymphocyte subsets and monocytes/macrophages was performed using the fluorochrome-conjugated antibodies. Cellular analysis data was collected using the Beckton Dickson LSR Fortessa flow cytometer (Franklin Lakes, NJ) and analyzed using FlowJo software (Tree Star Inc., Ashland, OR).

### IBA-1 immunohistochemistry and morphological analysis

Microglial activation in the hippocampus was examined using immunohistochemical localization of the microglial marker ionized calcium-binding adapter molecule 1 (IBA-1). Anesthetized fixed and shifted LD mice (*n* = 10; fixed LD: 2 females, 3 males; shifted LD: 2 females, 3 males) were immediately perfused transcardially with 50 ml of 0.1 M phosphate buffer (pH = 7.2). After perfusion, brains were removed and divided into hemibrains that were post-fixed in 4% paraformaldehyde for 48 h at 4^o^C and stored in 20% sucrose solution at 4^0^C until sectioning. Hemibrains were then frozen and sectioned serially through the entire hippocampus on a freezing microtome (coronal plane, 30 μm). Sections were stored in cryoprotectant solution (25% glycerin, 25% ethylene glycol, 50% 0.1 M phosphate buffer, pH 7.4) until subsequent immunohistochemical processing. With interceding rinses in Tris-buffered saline (TBS; 100 mM Tris-HCl, 150 mM NaCl, pH 7.5), free-floating sections were sequentially incubated in: blocking solution containing 10% bovine serum albumin (BSA) and 5% NGS normal goat serum (NGS) in TBS for 1 h, rabbit anti-IBA-1 pAb (1:2000; FUJIFILM Wako Pure Chemical Corp.) in TBS with 3% triton at 4^o^C for 48 h, and then Alexa Fluor 555-conjugated goat anti-rabbit IgG cross-absorbed secondary antibody (1:150; Invitrogen) in TBS for 2 h at room temperature in the dark. Sections were rinsed in TBS and mounted on Superfrost Plus slides (Fisher Sci.), air-dried at room temperature and coverslipped using Vectashield hardset antifade mounting medium with DAPI (Vector Labs). Slides were sealed with clear fingernail polish, and stored in the dark at 4°C.

High-quality images of microglial somas and cellular processes were acquired by confocal fluorescence microscopy (Olympus Fluoview FV3000) at maximum intensity projection (MIP). Using the 10X objective, images were captured from three representative sections for each animal (*n* = 10), and then three randomized fields of view (FOV) within the dentate gyrus (DG) of the hippocampus were analyzed in each section (9 DG per hemibrain). Within each FOV, individual microglia (approximately 10–20) in the DG were selected as regions of interest (ROI) according to the following criteria: (i) complete cell soma and processes and (ii) no overlap with other cells. The DG region was selected due to its involvement in memory consolidation [26, 27] and well-characterized localization of microglia [28, 29]. Next, 60X images were acquired by multi-place virtual Z-mode. On average the z-stack of each image was composed of 45–75 layers, depending on the Z-step size, which was optimized for each scan. Once the z-stack for randomized regions of interest was collected it was converted to a MIP format.

Manual morphological analyses were performed using FIJI software (Version1.54), specifically with the neuroanatomy and Sholl analysis plug-ins [30]. Before analysis, images were converted to 16-bit grayscale and binary processing was used to generate black and white images (Pixel radius: 3, mask weight: 0.6, radius: 1.0 pixel). Thresholds were manually adjusted between 15 and 20% depending on staining intensity. For each ROI, concentric circles with an increasing step size of 5 μm from the soma center were generated and then used to count the number of microglial processes at each radius length. In addition, ROIs were used to measure the soma area of each microglia and the integrated intensity of IBA-1 staining in the DG region.

Image acquisition and Sholl analysis were independently conducted by two investigators blinded to treatment groups. For each of the three FOVs in the DG of a given section, Sholl analysis was performed on ROIs encompassing approximately 10–20 cells, resulting in the morphological assessment of 90–180 IBA-1+ microglia per animal.

### Statistical analysis

The significance of LD treatment differences in circadian entrainment and quantitative parameters of the activity rhythm was determined by one-way ANOVA adjusted for multiple comparisons, followed by Tukey’s post-hoc pairwise analysis. For behavioral measures, statistical analyses were performed on the raw data using repeated measures ANOVA across days and one-way ANOVAs were performed on all other comparisons. For probe trials, the percent of the path (distance) in the target quadrant is used to quantify the animal’s insistence about the escape hole’s location. Quadrants contain five possible locations for escape, and the escape hole is in the middle of the target quadrant. Only data from the first 30 s are analyzed because mice typically give up searching after approximately 30 s. Group means from the probe trial were analyzed with one-way ANOVAs. Fisher’s PLSD post hoc analysis was used for more comprehensive Barnes maze analysis. Statistical analysis was performed on all flow cytometric data to determine the significance of LD treatment- and age-related differences using a one-way ANOVA adjusted for multiple comparisons in conjunction with Tukey’s post-hoc pairwise analysis. Pearson’s correlation coefficients were determined to analyze the relationship between the proportions of different subtypes of adaptive immune cells (B and T cells) in the spleen and cognitive index scores. In each case, LD treatment- and age-related differences in circadian entrainment parameters, cognitive behavior, adaptive immune cell profiling and IBA-1 immunostaining parameters were considered significant at *p* < 0.05 (GraphPad, San Diego, CA).

## Results

### Effect of shifted LD cycles on circadian rhythm of wheel-running activity

**Fig. 2:**
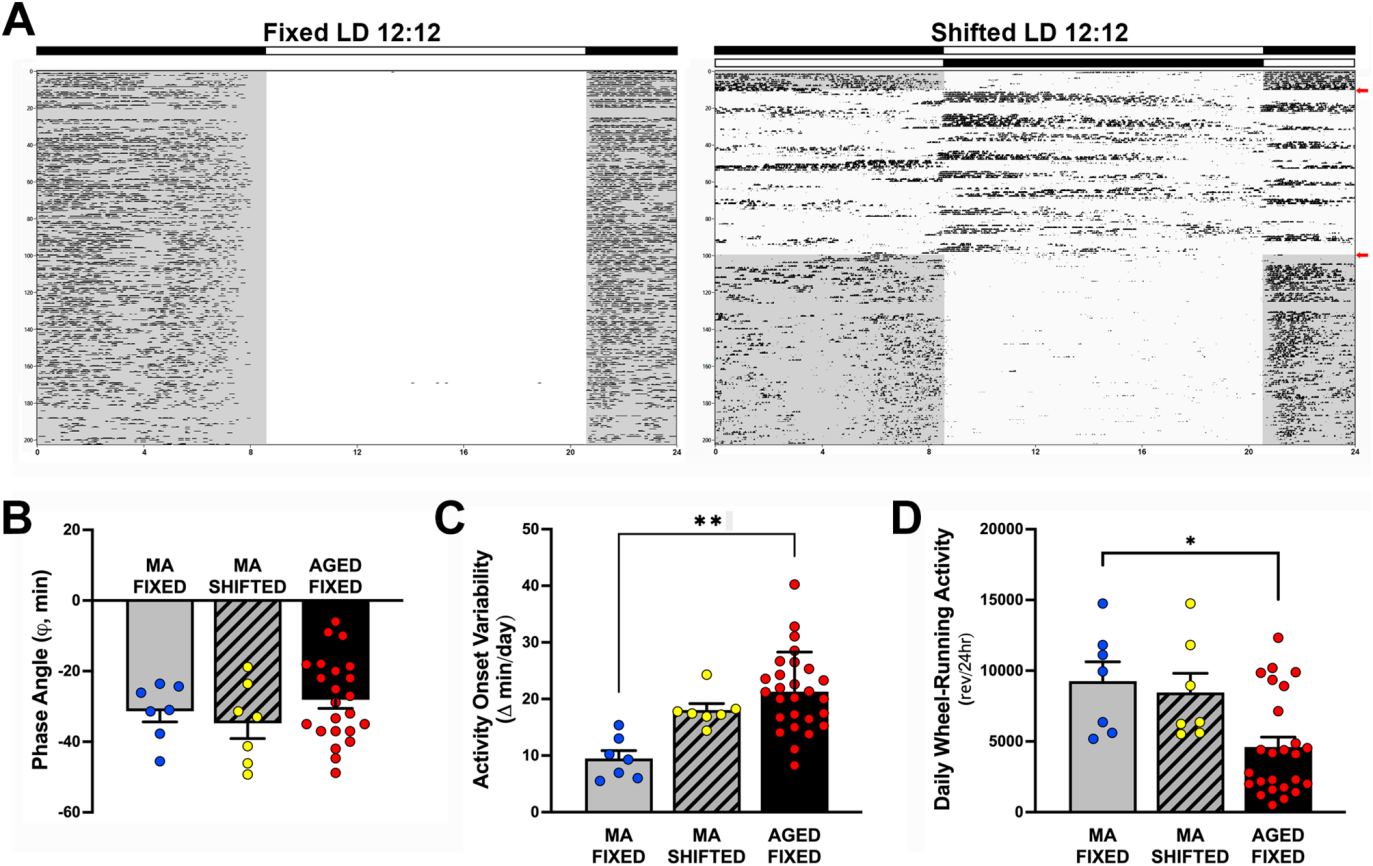
Effects of experimental LD cycles and aging on light-dark entrainment and other properties of the circadian rhythm in wheel-running activity. **(A)** Representative records of wheel-running activity in adult mice (≈ 3mo) that were maintained in a fixed LD 12:12 cycle (left) or exposed to a shifted (12 h/5d) LD 12:12 cycle (right). Actograms are plotted over a 24-hour period. The open and closed bars at the top respectively signify the timing of the light and dark phase in the fixed and shifted LD 12:12 cycles. Red arrows on the right denote the interval when exposure to the shifted LD cycles was initiated (“treatment” phase) and when shifted LD animals were returned to the same regular LD 12:12 schedule as the fixed LD group (post-treatment phase). **(B)** The phase angle (Ψ) between daily activity onsets and lights-off, **(C)** absolute day-to-day variability, and **(D)** total daily wheel-running activity (wheel revolutions/24hr) were later analyzed during the post-treatment phase in fixed (*n* = 7) and shifted (*n* = 7) LD mice at middle age (13-14mo). Then these entrainment and qualitative parameters of the activity rhythm in middle-aged mice from both treatment groups were compared to similar published data obtained from aged mice on fixed LD cycles (Souza et al., [14]). In panel **B**, negative phase angle values (in minutes) indicate that daily onsets of activity occur after lights-off. Bars (in **B**-**D**) depict mean values (+ SEM). Circles indicate individual data values for each mouse. (*p < 0.01; Tukey’s multiple comparisons)

During baseline acclimation to the standard LD 12:12 cycle, stable entrainment of the circadian rhythm of wheel-running activity was observed in all animals. Throughout the period of exposure to experimental lighting conditions, the activity rhythms of all mice in the fixed LD group remained stably entrained to the LD 12:12 cycle (Fig. 2A, left panel), with daily onsets of activity occurring shortly after lights-off (2000 h). In contrast, mice exposed to shifted LD cycles (Fig. 2A, right panel) were distinguished by desynchronized rhythms of wheel-running behavior, such that the phase relationship between the onset of activity and lights-off was highly variable after each shift of the LD cycle. When both groups were exposed to the same LD 12:12 schedule (lights-on at 0800 h) for the remainder of the experiment, photoentrainment of the activity rhythm was sustained in fixed LD animals and was reinstated in the shifted LD group. Immediately after experimental LD cycle manipulations, the pattern of entrainment was virtually the same in fixed LD mice with stable alignment of activity onsets shortly after lights-off but was altered in the shifted LD group such that day-to-day variability in activity onset times was greatly increased (Fig. 2A). However, initial differences between fixed and shifted LD mice in this and other circadian parameters of the activity rhythm receded at middle age. No significant differences in the phase angle of entrainment (ϕ), daily activity onset variability or daily activity levels (wheel revolutions/24hr) were observed between middle-aged (MA) fixed and shifted LD mice (Fig. 2B-D). Further comparison with our published data from aged C57Bl/6 mice exposed to fixed LD cycles [14] was used to discriminate between the effects of normal aging and circadian dysregulation in response to shifted LD cycles on these key parameters of light-dark entrainment. The middle-aged fixed, middle-aged shifted and aged fixed LD cohorts exhibited phase angles of entrainment in which their daily onsets of activity were delayed relative to young mice (data not shown) and occurred at later times, commencing up to 30–45 min after lights-off (Fig. 2B). Day-to-day variability in activity onset times of the middle-aged fixed, middle-aged shifted and aged fixed LD groups tended to increase with age and this index of labile entrainment was significantly greater (F(3,55) = 34.90, *p* < 0.01) in aged than middle-aged mice maintained on fixed LD cycles (Fig. 2C). Group comparisons of middle-aged fixed, middle-aged shifted and aged fixed LD animals also revealed a general age-related decline in the total amount of daily wheel-running activity, with middle-aged mice showing significant decreases (F(3,52) = 22.68, *p* < 0.05) in daily activity (wheel revolutions/24hr) relative to the levels observed in their aged counterparts exposed to fixed LD cycles (Fig. 2D).

### Comparison of the long-term effects of shifted LD cycles and aging on cognitive performance in the Barnes maze

**Fig. 3:**
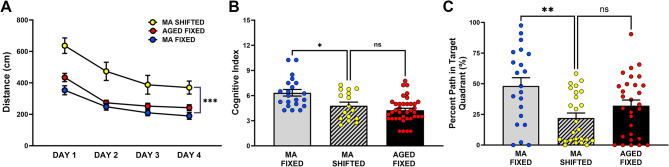
Effects of experimental LD cycles on cognitive performance in the Barnes maze. **(A)** Distance traveled (cm) to reach the escape is depicted across days of learning trials, **(B)** cognitive index for training trials, and **(C)** percent path in target quadrant (i.e., quadrant in which the escape was localized during training trials, during the first 30 s of the trial) were analyzed in fixed (*n* = 20) and shifted (*n* = 16) LD mice at middle age (13-14mo) and compared to similar data from aged mice on fixed LD cycles (Souza et al., [14]). Plotted values (in **A**-**C**) represent mean ± SEM. Circles (in **B**-**C**) indicate individual data values for each mouse. (*p < 0.05, **p < 0.01; ***p < 0.0001; Fisher’s PLSD post hoc analysis)

Based on our previous findings that pronounced alterations in circadian rhythm entrainment (i.e., delayed phase angle and increased variability in the daily onsets of activity) first occur in middle-aged mice preceding age-related deficits in learning and memory [14], we compared the cognitive performance of middle-aged mice exposed to shifted LD cycles during early adulthood to the same measures for middle-aged and aged cohorts of fixed LD controls. At middle age, shifted LD mice were distinguished from all age groups of fixed LD animals by a dramatic increase in their distance to reach the escape box throughout the 4 days of learning (Fig. 3A). For these distance comparisons, there was an overall effect between groups (F(3,72) = 21.533, *p* < 0.0001) and all groups performed better over time (F(3,186) = 32.382, *p* < 0.0001). Post-hoc analysis with Fisher’s PLSD indicates that the middle-aged mice exposed to shifted LD cycles took significantly longer (distance in cm) paths (*p* < 0.0001) when compared to their fixed LD counterparts at middle age or even to our previously reported data [14] from aged cohorts of fixed LD controls (Fig. 3A). Our previous study [14] established that cognitive index scores provide a reliable gage of hippocampal search strategies in the Barnes maze, with aged mice showing lower cognitive indices (mean = 4.431) than middle-aged animals exposed to fixed LD cycles (mean = 6.250). In the present study, overall differences in cognitive index were observed between groups (F(2, 72) = 11.34, *p* < 0.0001; Fig. 3B). Multiple comparisons of the search strategy during learning revealed that middle-aged mice exposed to a shifted LD cycles during early adulthood showed significant decreases (*p* < 0.05) in cognitive index (mean = 4.802) relative to middle-aged mice maintained on fixed LD cycles (mean = 6.333). However, no significant differences in cognitive index (*p* = 0.489) were observed between middle-aged mice exposed to shifted LD cycles and aged fixed LD animals (mean = 4.25). These results demonstrate that both aged fixed LD mice and middle-aged shifted LD mice are distinguished by a decrease in use of hippocampal-dependent strategies, which are normally preferred by healthy young [14] and non-impaired middle-aged controls on fixed LD cycles. Similar shortcomings in group comparisons were apparent in the memory portion of the task, when assessing the percentage of path in the target quadrant during the probe trial (Fig. 3C). Group differences were detected in this measurement (F(2,72) = 6.201, *p* < 0.01), and pathlength concentration in the target quadrant was significantly decreased (*p* < 0.01) in the shifted LD group relative to their middle-aged counterparts exposed to fixed LD cycles.

### Long-term effects of circadian dysregulation on adaptive immune cells in the spleen

**Fig. 4:**
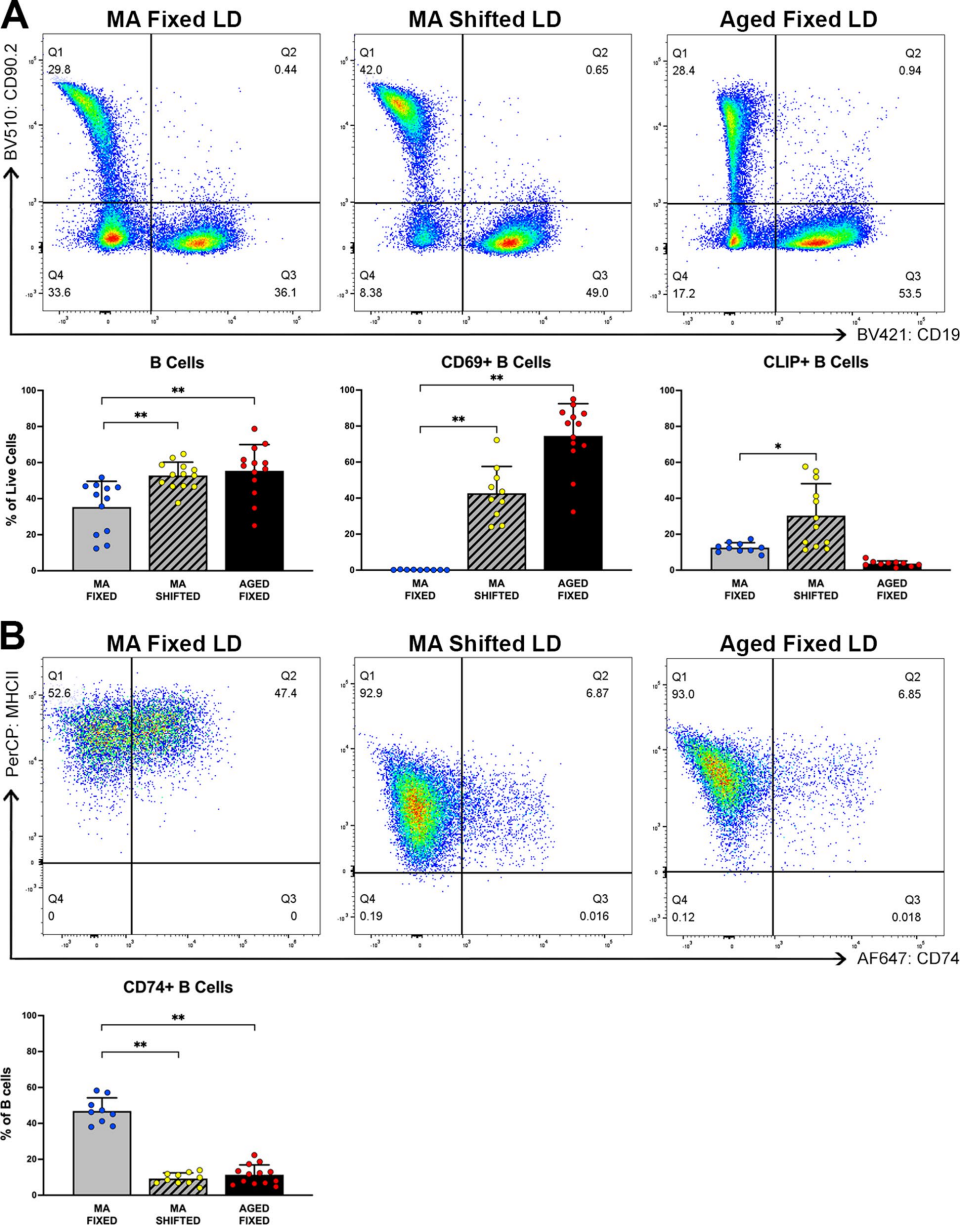
Effects of experimental LD cycles and aging on splenic B cell populations. (**A**) Representative dot plots (top) using CD19 (x-axis) as a B cell marker versus CD90.2 as a T cell marker to compare populations of B cells in isolated splenocyte samples from middle-aged (MA) mice on fixed (left) and shifted (12 h/5d, center) LD 12:12 cycles, and from an aged (18-22mo) animal (right) maintained on a fixed LD cycle. Bar graphs (bottom) depict group comparisons (MA fixed, *n* = 12; MA shifted, *n* = 13; Aged fixed, *n* = 13) of the percentage (mean ± SEM) of B cells identified from Quadrant 3 (left), and of CD19+ B cell populations expressing the activation marker CD69 (center) or cell surface CLIP (right). (**B**) Representative dot plots (top) of live B cells gated from quadrant 3 (see panel **A**) displaying MHCII (y-axis) and CD74 (x-axis) expression. Bar graph (bottom) depicts group comparisons (MA fixed, *n* = 9; MA shifted, *n* = 10; Aged fixed, *n* = 10) of the percentage (mean ± SEM) of CD74+ B cells identified from quadrant 2 and 3 (left). (*p < 0.05, **p < 0.01; Tukey’s multiple comparisons)

Comparisons of flow cytometric data in middle-aged mice revealed that exposure to shifted LD cycles during early adulthood induced an overall expansion of B cells in the spleen when compared to those found in fixed LD mice. In middle-aged shifted as well as aged fixed LD mice, the percentage of B cells in the spleen was significantly increased (F(6,76) = 10.27, *p* < 0.01) relative to that found in fixed LD mice at middle age (Fig. 4A). Consistent with these LD cycle treatment-induced differences in relative percentage, the total number of splenic B cells was increased by ≈ 1.4-fold in shifted LD mice when compared to fixed LD controls at middle age. Coupled with the increase in total number of B cells, the middle-aged cohorts of fixed and shifted LD mice exhibited a variety of differences in splenic B cell subtypes. At middle age, the percentage of CD19+ B cells expressing the activation marker CD69 on the expanding B cells from the spleen was significantly increased (F(5,49) = 45.15, *p* < 0.01) in shifted LD mice relative to that found in fixed LD controls (Fig. 4A). In middle-aged mice exposed to shifted LD cycles, the expansion of splenic B cells was also accompanied by a significant increase (F(5,58) = 9.537, *p* < 0.05) in the percentage of CD19+ B cells that express cell surface CLIP + relative to that found in age-matched mice on fixed LD cycles (Fig. 4A). In contrast to the increase in CD69+ B cells, the percentage of splenic populations of CD19+ B cells expressing CD74+ in middle-aged mice exposed to shifted LD cycles was significantly reduced (F(4,42) = 77.46, *p* < 0.01) in comparison with that observed in the middle-aged cohort of fixed LD controls (Fig. 4B). Furthermore, the percentage of CD74+ B cells differed across age groups of fixed LD mice, such that this subpopulation of B cells was significantly reduced (*p* < 0.01) in the aged cohort compared to middle-aged mice (Fig. 4B). One possible explanation for these results is that CD74 is known to be internalized following its interaction with its ligand, the pro-inflammatory cytokine MIF [31, 32], and then newly internalized CD74 may be proteolytically cleaved into its peptide product CLIP and loaded into the peptide binding groove of the MHC Class II molecule.

**Fig. 5:**
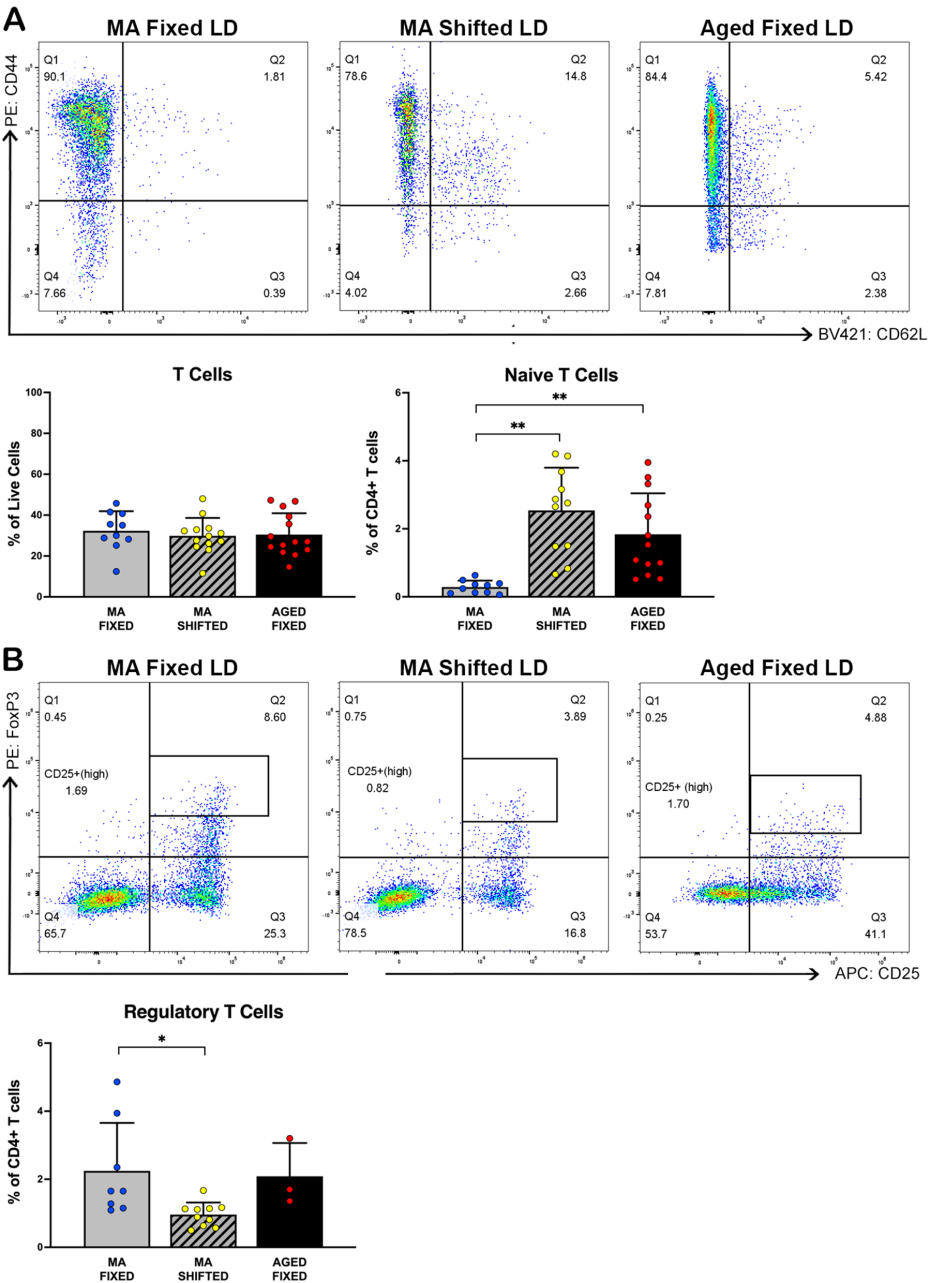
Effects of experimental LD cycles and aging on splenic T cell populations **(A)** Flow cytometry analysis using CD90.2 (y-axis from Fig. 4A, quadrant 1) as a T cell marker to compare populations of T cells in isolated splenocyte samples from middle-aged (MA) mice on fixed (left) and shifted (12 h/5d, center) LD 12:12 cycles, and from an aged (18-22mo) cohort (right) of fixed LD mice. Representative dot plots (top) depict gated CD4+ T cells exhibiting expression of CD44 (y-axis) and CD62L (x-axis). Bar graphs (bottom) depict group comparisons (MA fixed, *n* = 10; MA shifted, *n* = 11–13; Aged fixed, *n* = 13–14) of the percentage (mean ± SEM) of T cells (left) identified in quadrant 1 of Fig. 4A and of CD4+ naïve T cells (right) that were gated from quadrant 3 as CD62L+/CD44-. **(B)** Representative dot plots (top) of CD4+ T cells displaying expression of FoxP3 (y-axis) and CD25 (x-axis). FoxP3 + and CD25+ (high) expression was used to gate regulatory T cells, as shown in the outlined box. Bar graph (bottom) depicts group comparisons (MA fixed, *n* = 8; MA shifted, *n* = 10; Aged fixed, *n* = 3) of FoxP3+ and CD25+ regulatory T cells. (*p < 0.05, **p < 0.01; Tukey’s multiple comparisons)

In contrast to the observed expansion of splenic B cells, the percentage of T cells in the spleen of middle-aged mice exposed to shifted LD cycles during early adulthood was not significantly different (F(6,65) = 3.249, *p* = 0.992) from that found in age-matched fixed LD controls (Fig. 5A). However, shifted LD mice at middle age were distinguished by alterations in various sub-populations of splenic T cells, including their activation, effector status, and regulatory T cell phenotypes. Gating was performed to compare CD4+ T cell subsets with regard to the expression of CD44 and CD62L, which can be used to distinguish memory, effector, and naïve populations of CD4+ T cells [33]. The analysis of CD4+ T cell phenotypes revealed that the percentage of naïve CD62L+/ CD44- T cells in the spleen of shifted LD mice at middle age was significantly increased (F(6,64) = 10.94, *p* < 0.01) in comparison with their age-matched counterparts maintained on fixed LD cycles (Fig. 5A). One possible explanation for this observation is that the increased percentage of this T cell subset may be the consequence of a corresponding decline in a different subset (i.e., Tregs; Fig. 5B), which would account for the upward shift in percentage of naïve T cells. In turn, this decline in number could possibly stem from either T cell migration out of the spleen, T cell “exhaustion”, or cell death of more activated T cells. In addition, age-related differences were observed in the splenic populations of these T cell phenotypes such that in fixed LD mice, naïve CD62L+/CD44- T cells were significantly increased (*p* < 0.01) in the aged cohort compared to middle-aged mice (Fig. 5A). Consistent with the potential relationship between populations of naïve and regulatory T cells, gating of live CD4+ cells using the extracellular markers CD4 and CD25, and intracellular marker FoxP3 (Fig. 5B) revealed that the percentage of FoxP3+/CD25+ (high) regulatory T cells was significantly decreased (F(3,21) = 3.442, *p* < 0.05) in the spleen of shifted LD mice relative to their middle-aged counterparts exposed to fixed LD cycles. These results are noteworthy because a similar reduction in regulatory T cells has been reported in multiple models of autoimmune disease, including juvenile idiopathic arthritis, psoriatic arthritis, hepatitis C virus (HCV)-associated mixed cryoglobulinaemia, autoimmune liver disease, systemic lupus erythematosus^,^ and Kawasaki disease [34]. No significant differences in splenic populations of FoxP3+/ CD25+(high) regulatory T cells (*p* = 0.86) were observed between the middle-aged and aged groups of fixed LD mice.

**Fig. 6:**
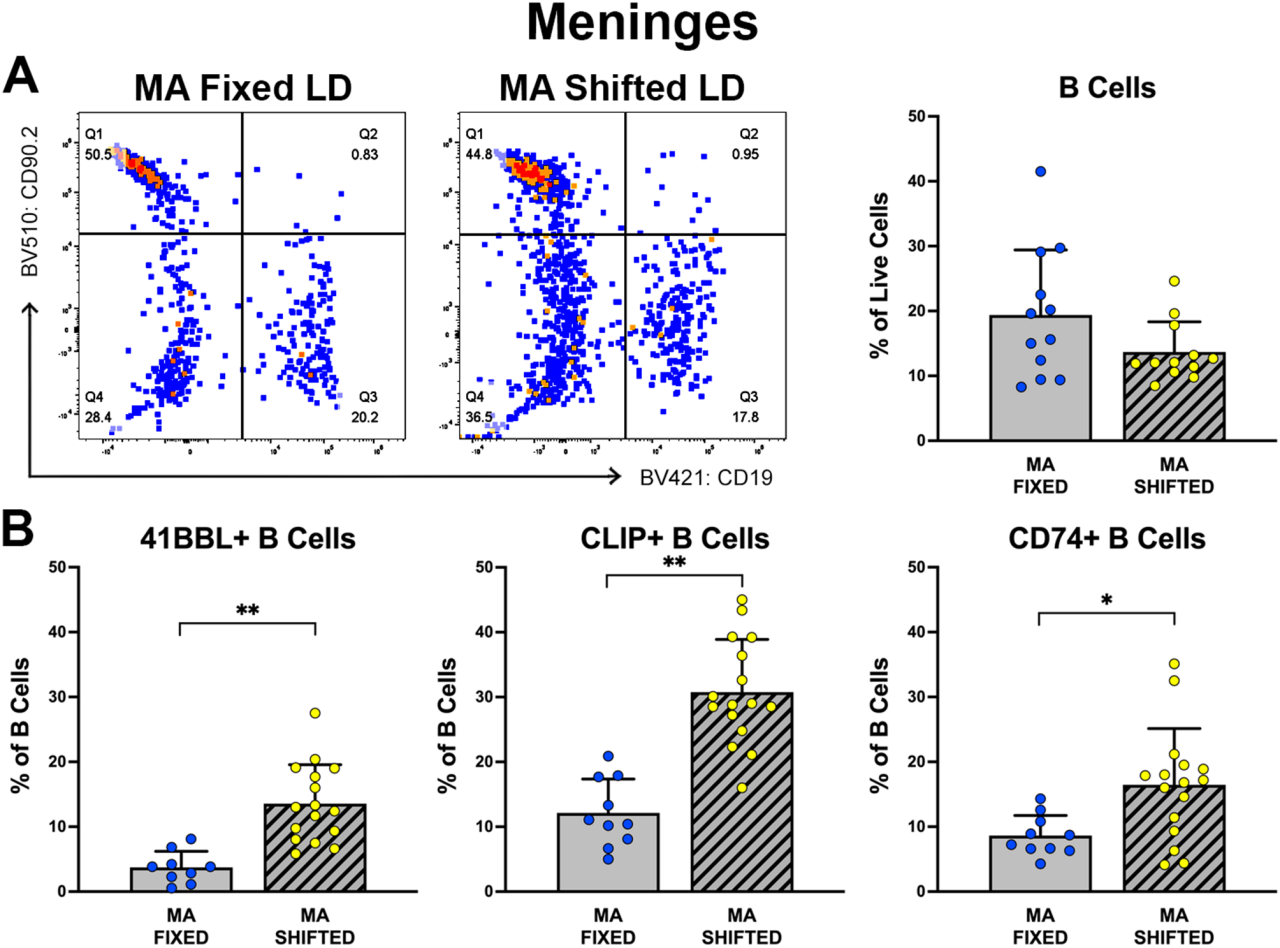
Effects of experimental LD cycles on meningeal B cell populations. (**A**) Representative dot plots using CD19 (x-axis) as a B cell marker versus CD90.2 as a T cell marker to compare populations of B cells in isolated meningeal samples from mice on fixed (left) and shifted (12 h/5d, right) LD 12:12 cycles. Bar graph depicts group comparisons of the percentage (mean ± SEM) of CD19+ B cells (right). **(B)** Bar graphs depict group comparisons (MA fixed, *n* = 9–10; MA shifted, *n* = 16) of the percentage (mean ± SEM) of CD19+ B cell subsets expressing 41BBL (left), CLIP (center), or CD74 (right) as indicated. (*p < 0.05, **p < 0.01; Mann-Whitney test)

Because the progressive cognitive decline during aging and in ADRDs is associated with central nervous system (CNS) inflammation [35], lymphocytes from the cranial meninges were analyzed in parallel to determine whether shifted LD cycles similarly have long-term effects on adaptive immune cells in the brain. Although the percentages of CD19+ B cells in the cranial meninges were not significantly different (*p* = 0.183) between fixed and shifted LD mice at middle age (Fig. 6), circadian dysregulation during early adulthood induced long-term alterations in meninges-derived populations of B cell subtypes. At middle age, the proportions of meningeal CD19+ B cells expressing the activation marker 41BBL, CD74 or CLIP in shifted LD mice were significantly increased (*p* < 0.01) relative to those observed in fixed LD controls, indicating that circadian dysregulation promotes long-term expansion and activation of a more pro-inflammatory subset of B cells in both the brain and periphery.

**Fig. 7:**
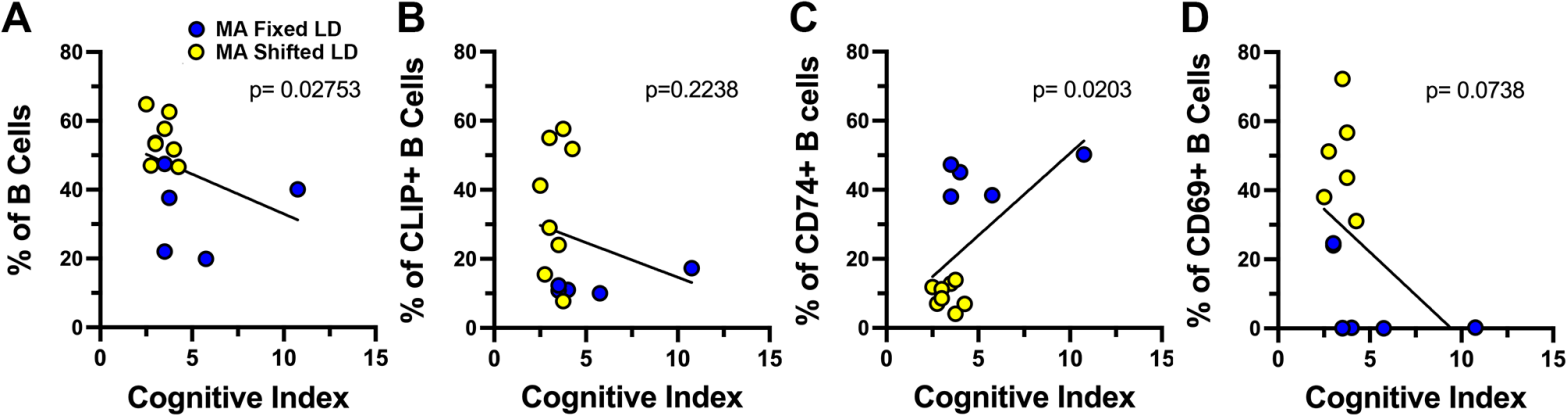
Relationship between splenic B cell populations and cognitive performance in Barnes maze for middle-aged (MA) mice that were exposed to fixed or shifted LD cycles. Pearson correlation coefficients comparing cognitive index with the percentage of: (**A**) B cells, (**B**) CLIP + B cells, (**C**) CD74 + cells and (**D**) CD69 + cells in the middle-aged cohorts of fixed (*n* = 5) and shifted (*n* = 6–8) LD mice. Circles represent individual data values for each mouse. Lines in each graph denote simple linear regression for the data set with corresponding *p* values

Further analysis of our flow cytometry data using Pearson correlation coefficients revealed an interesting relationship between splenic B cell populations and cognitive function in response to shifted LD cycles. Because it represents a single value determination of individual cognitive performance as commonly assessed in human clinical studies, the cognitive index (CI) of fixed and shifted LD mice at middle age was used to compare the extent of impaired learning with the proportions of different B cell populations for each animal. The total number of B cells and the percentage of CD74+ B cells in the spleen were directly associated with CI scores in individual animals. A one-tailed correlational analysis with a confidence interval of 95% revealed a significant negative correlation between CI and the percentage of total splenic B cells (*p* = 0.027, *R*=-0.55, Fig. 7A). However, CI was positively correlated with the proportion of CD74+ B cell (*p* = 0.020, *r*=-0.57, Fig. 7C) populations, such that when the splenic population of this B cell subtype was high, scores were in the upper range and performance was correspondingly better in the Barnes maze. In contrast, the opposite trend toward a negative relationship was observed between CI and the percentage of CD69+ and CLIP+ B cells in the spleen. Animals with low CI scores were characterized by higher percentages of CLIP+ (*p* = 0.224, *r*=-0.231, Fig. 7B) and CD69+ (*p* = 0.074, *r*=-0.425, Fig. 7D) B cells.

### Long-term effects of circadian dysregulation on hippocampal microglia

**Fig. 8:**
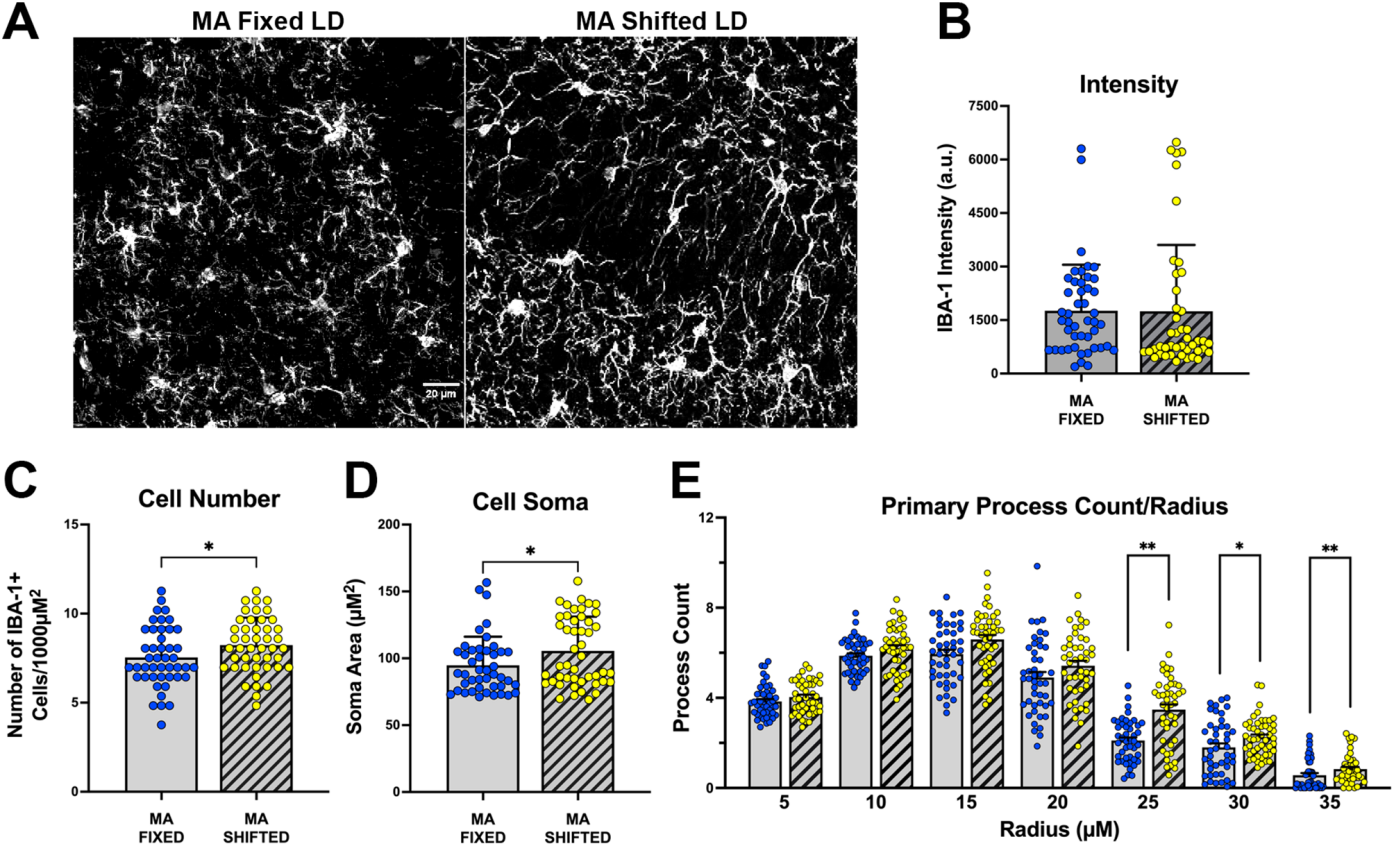
Effects of experimental LD cycles on the morphology of hippocampal microglia. (**A**) High-magnification (60X) confocal images of IBA1+ hippocampal microglia located within the dentate gyrus (DG) of representative fixed (left) and shifted (right) LD mice at middle age. Bar graphs depict quantitative analysis of the morphological profiles of IBA-1+ microglia in the DG (mean ± SEM) from regions of interest (ROI) in fixed (*n* = 5) and shifted (*n* = 5) LD mice with regard to: (**B**) integrated fluorescent intensity, (**C**) number of immunopositive cells, (**D**) soma area, and (**E**) the number of primary immunopositive processes with Sholl intersections (at 5 μm intervals of the distance from the microglial soma). For all morphological analyses, three representative hemibrain sections through the hippocampus were analyzed from each animal and three randomized FOV within the dentate gyrus (DG) of the hippocampus were captured in each section. In turn, the data points represent individual IBA-1+ microglia (approximately 10–20/FOV) in the DG that were selected as ROI. (*p < 0.05, **p < 0.01; Mann-Whitney test)

In addition to the increases in inflammatory B cell types and decrease in anti-inflammatory immune cells, middle-aged mice exposed to shifted LD cycle during early adulthood exhibited clear differences in the morphology of microglia within the DG of the hippocampus relative to age-matched fixed LD controls. IBA-1+ microglia were scattered throughout the hippocampus of both fixed and shifted LD mice and immunofluorescence was localized in the cell soma of microglia and their processes extending into the surrounding parenchyma. In middle-aged mice exposed to shifted LD cycles, the soma of microglia in the DG were more amoeboid-shaped and somewhat enlarged with extensive reticular arborization of their processes relative to those observed in the middle-aged cohort of fixed LD controls (Fig. 8A). Based on morphological analysis, the integrated intensity of immunofluorescence in the DG was not significantly different (*p* = 0.1452; Fig. 8B) between middle-aged fixed and shifted LD mice. However, further comparisons of middle-aged cohorts indicated that both the number and cell soma area of IBA-1+ microglia in the DG of LD shifted mice were significantly increased (*p* < 0.05) relative to the values found in fixed LD controls (Fig. 8C and D). Sholl analysis of the number of process intersections at 5µM increments from the cell soma (Fig. 8E) revealed further differences in the morphological state of microglia in the DG of middle-aged fixed and shifted LD mice; the number of IBA-1+ microglia in the DG with process intersections at radius distances of 25, 30 and 35µM from the cell soma was significantly greater (*p* = 0.0001, *p* = 0.0344, and *p* = 0.0099, respectively) in shifted LD animals than in fixed LD controls. Overall, these increases in microglia number, soma area and process length in shifted LD mice suggest that circadian dysregulation during early adulthood induces long-term alterations in the functional state of microglia in the hippocampus.

## Discussion

Desynchronization of cell-specific circadian clocks and dysregulation of their output rhythms in response to irregular daily schedules associated with shift work and workplace or social influences have been implicated in cognitive health. Recent epidemiological studies indicate that circadian rhythm misalignment adversely affects cognitive function in shift workers [36–38]. However, observations from these studies provide limited opportunity to distinguish the long-term pathological impact of circadian dysregulation alone from aging and other risk factors for dementia. Using a chronic LD cycle shifting paradigm, the present study demonstrates that early circadian dysregulation, even with intervening entrainment to a stable LD cycle for at least 7 months, induces dramatic cognitive impairment at middle age, well before learning and memory deficits normally occur in C57Bl/6J mice. Importantly, these findings establish for the first time that circadian rhythm desynchronization/misalignment, by itself, is a long-term risk factor for accelerated cognitive decline later in life, suggesting that dysregulation of circadian timekeeping is prodromal to cognitive aging.

Individual differences in the extent of age-related cognitive and neuronal dysfunction are well documented in humans and rodent models [39]. Across species, some aged subjects show no significant decline in cognitive function and perform within the range of their younger counterparts demonstrating a resilience to the effects of aging, whereas others are distinguished by pronounced cognitive impairment [40, 41]. Differences in the extent of cognitive aging may be related to genetics and disease-specific pathology, or alternatively to other lifestyle variables, such as diet and sleep-wake patterns. Implications of the relationship between circadian dysregulation and cognitive aging were fostered by our previous study where only 55% of the aged mice (18-22mo) were impaired learners but all in this subgroup were differentiated from the remaining aged subjects with unimpaired cognitive performance by altered and unstable patterns of circadian entrainment [14]. In fact, learning-impaired mice in all age groups were distinguished by a negative correlation between day-to-day variability in the onsets of circadian activity and CI scores on the Barnes maze. The link between altered circadian entrainment and cognitive impairment in aging is further reinforced by the current findings that controlled circadian dysregulation during early adulthood induced dramatic impairment on the Barnes maze test in the entire group (100%) of shifted LD mice at middle age. Collectively, these studies suggest that stable regulation and alignment of circadian rhythms may be key factors in the resilience to age-related cognitive decline.

Central nervous system (CNS) inflammation underlying cognitive and behavioral impairments in aging and AD [35] is characterized by a pathophysiological cascade, including the activation of adaptive immune cell subpopulations, that contributes to synaptic and neuronal loss. In particular, changes in B cell [42, 43] and T cell [44, 45] subtypes, coupled with elevated levels of pro-inflammatory cytokines [46] have been linked to neuroinflammation, age-related cognitive decline and AD pathophysiology [19–22]. Similar to the implications of immune cell activation and inflammation in cognitive impairment during aging, circadian rhythm dysregulation has been shown to promote pro-inflammatory responses by the immune system, leading to a persistent inflammatory condition [17, 18, 23, 47]. Likewise, circadian misalignment in shift workers has been associated with key alterations in immune/inflammatory processes [48], such as changes in adaptive immune parameters contributing to increased vulnerability to severe CoVID19 disease [37, 49]. Consistent with the reported commonality of altered activation of adaptive immune cells in both circadian dysregulation and cognitive aging, novel observations in this study indicate that early exposure to shifted LD cycles promotes expansion of B cells, as well as inflammatory CLIP + B cells, in the spleen of middle-aged mice. In the meninges, while an expansion in the total number of B cells was not observed, there was a significant increase in the percentage of B cells expressing pro-inflammatory markers, including 41BBL, CLIP, and CD74. These findings are compatible with evidence for increased populations of both B cells and CLIP+ B cells in neuroinflammatory pathologies [50, 51]. Considering the role of B cells in: (1) preserving the antigen specificity of antibody-mediated adaptive immune responses; (2) producing pro-inflammatory or anti-inflammatory cytokines that regulate innate immune responses underlying adaptive immunity; and (3) acting as efficient antigen presenting cells (APC) that convey antigens associated with Major Histocompatibility Complex (MHC) Class II molecules leading to T cell activation and cytokine production [50], these data support the hypothesis that circadian dysregulation promotes expansion of pro-inflammatory B cells in both the periphery (spleen) and the central nervous system (dural meninges) that may contribute to the inflammation associated with accelerated cognitive aging.

In addition to its effects on B cells, circadian dysregulation in response to shifted LD cycles during early adulthood induced a concomitant reduction in splenic regulatory T cells at middle age. In this context, it is noteworthy that regulatory T cells mediate immunosuppression and provide protection against autoreactivity [52–54], and that decreases in this adaptive immune cell type have been implicated in autoimmune and/or chronic inflammatory conditions, and in cognitive decline during aging [21, 22]. Importantly, circadian dysregulation-induced expansion of pro-inflammatory B cell subtypes in the meninges was also coupled with further signs of neuroinflammation in the brain as suggested by long-term alterations in the morphological state of hippocampal microglia in mice exposed to shifted LD cycles during early adulthood. The implications of this altered state and the increased number of hippocampal microglia in the accelerated cognitive impairment in shifted LD mice at middle age are consistent with the putative contributions of these resident innate immune cells in neuroinflammatory processes associated with the pathophysiology of age-related cognitive decline and ADRDs [55], including the spread of tau pathology [56].

Although the specific mechanism by which disordered circadian timekeeping associated with irregular work or social schedules contributes to cognitive impairment during aging is unknown, these findings collectively suggest that innate or adaptive immune cells, microglia in the brain and their inflammatory responses may play a critical role in mediating the pathological effects of circadian rhythm dysregulation. In future studies, it will be necessary to determine whether treatment strategies blocking the expansion of B cells, or selectively targeting a pro-inflammatory subset of B cells, and/or perhaps augmenting regulatory T cell populations, may be useful in abrogating the effects of circadian dysregulation in activating inflammatory immune cells and accelerating age-related cognitive impairment.

## Data Availability

No datasets were generated or analysed during the current study.
